# Pension Problems

**Published:** 1917-01-20

**Authors:** 


### NATIONAL INSURANCE ACT DEVELOPMENTS.

### Pension Problems.

It may, perhaps, appear that the grant of pensions to disabled soldiers has no connection with National Health Insurance, but investigation reveals the fact that approved societies are seriously concerned with the question as referring to their members.

When an insured man joins the Army he is, for the duration of his service with the Colours, grouped as Class B for national insurance purposes. That is to say, a reduced contribution is paid in respect of him, and correspondingly he loses, for the time being, his right to sickness and disablement benefits. Upon his discharge, however, the reduced contributions which have been paid during his period of service ensure to him the right to claim the ordinary sickness and disablement benefits from the approved society, of which he has all the while remained a member, whilst rendered incapable of work by some specific disease or by bodily or mental disablement.

Quite early in the duration of the war it was realised that, as the contingency of a great European conflict had not entered into the calculations upon which the finance of the National Insurance Act was framed, something would have to be done to relieve approved societies of the unexpectedly serious strain that would otherwise have been imposed upon their funds. Thus in March 1915 an Act was passed, under the title National Insurance (Part I. Amendment) Act, 1915, by virtue of which it is provided that when upon his discharge a pension in respect of *total* disablement suffered in consequence of the present war is granted to a disabled soldier, the amount of any sickness or disablement benefit to which he may be entitled is subject to a reduction of five shillings a week so long as he continues in receipt of such total disablement pension. Seeing that disablement benefit is itself five shillings a week, the effect of the Amendment Act was to relieve the approved societies of all payments in respect of incapacity of long duration.

The Amendment Act, however, went on to provide that in the case of pensions, other than total disablement pensions, granted under certain specified Acts, which for convenience may be described as partial disablement pensions, the amount of such pension is to be treated in the same manner as compensation received under the Workmen's Compensation Act. That is to say, the approved societies were intended to be put under the obligation of making good any sum by which the pension falls short of the sickness or disablement benefit as the case may be.

### Partial Disability Pensions.

At the time the Amendment Act was passed it appeared to meet with general approval, but as the result of actual experience the position is not now considered to be satisfactory by the approved societies. The reason is that the vast majority of the pensions granted have been given as partial disablement pensions, and not as total disablement allowances, with the result that the Act has not brought the measure of relief which approved societies had anticipated. The large and increasing number of such cases is giving rise to some uneasiness on the part of approved society officials, and they have endeavoured to secure the relief desired by application to the Insurance Commissioners. The power to give such relief not being vested in the Commissioners, a number of approved societies have gone so far as to indicate that reliance will have to be placed upon the official description of the pension granted. Thus, if a pension is stated to be a *partial* disablement pension, it is urged that the very words employed indicate that the discharged soldier is not rendered absolutely incapable of work by the disablement on account of which the pension was granted. In other words, these societies contend that, if the award of the competent pension authority made, presumably, after full investigation of the facts is a partial disablement pension it would not be feasible to allow the claim of the man that he was in fact totally incapable of work.

### Is Work Reasonably Available?

There is a good deal to be said for the argument, but it would probably never have been raised if the number of claims had been small. The very fact that it has been used is indicative of the inroad which the claims are making on the funds of the societies; for the societies are desirous of acting in the interests of their members, and would not without good reason put forward any purely technical plea, especially when the members concerned are disabled soldiers.

The matter has evidently been brought prominently to the notice of the Insurance Commissioners, for they have recently issued a special circular (A.S. 183) in which they deal with it at some considerable length. It is doubtful, however, whether the view of the Commissioners will facilitate in any marked degree the settlement of the difficulty. That view is "that an insured person, who, though incapable of following his ordinary occupation, is yet physically capable of doing work of some kind cannot be regarded as incapable of work, . . . if such work is *reasonably available to him*, due regard being had to his training, aptitude and other circumstances; but if, on the contrary, such work is *not reasonably available* he should be considered as incapable of work."

This is, of course, duly elaborated, but so much depends upon the interpretation of the words which we have italicised that it is doubtful whether any two societies would adopt exactly similar lines in dealing with any given number of actual cases.

So much is left to the exercise of personal opinion, and this cannot be considered satisfactory in dealing with a problem of the magnitude of that which is here involved.

The Commissioners are on safer and more practical ground when they suggest to approved societies that with a view to obtaining the relief to which they are entitled, under the Act of 1915, they should, whenever it appears to them that a *prima facie* case can be made out for the reconsideration of the award of pensions, assist their members by approaching the appropriate pension authority.

They also point out that an object to be borne in mind is to prevent a disabled soldier from drifting into habits of idleness, and societies are recommended to bring to the notice of the local War Pension Committee any cases where that authority might be able to find work for the man.

